# TNFα-mediated activation of NF-κB downregulates sodium-iodide symporter expression in thyroid cells

**DOI:** 10.1371/journal.pone.0228794

**Published:** 2020-02-12

**Authors:** Márcia Faria, Rita Domingues, Francisca Paixão, Maria João Bugalho, Paulo Matos, Ana Luísa Silva

**Affiliations:** 1 Serviço de Endocrinologia, Diabetes e Metabolismo do CHULN-Hospital Santa Maria, Lisboa, Portugal; 2 BioISI-Biosystems and Integrative Sciences Institute, Faculdade de Ciências da Universidade de Lisboa, Lisboa, Portugal; 3 Departamento de Genética Humana, Instituto Nacional de Saúde Doutor Ricardo Jorge, Lisboa, Portugal; 4 ISAMB-Instituto de Saúde Ambiental, Faculdade de Medicina da Universidade de Lisboa, Lisboa, Portugal; 5 Faculdade de Medicina da Universidade de Lisboa, Lisboa, Portugal; University of Leeds, Faculty of Medicine and Health, UNITED KINGDOM

## Abstract

The sodium-iodide symporter (NIS) mediates transport of iodide across the basolateral membrane of thyroid cells. NIS expression in thyroid cancer (TC) cells allows the use of radioactive iodine (RAI) as a diagnostic and therapeutic tool, being RAI therapy the systemic treatment of choice for metastatic disease. Still, a significant proportion of patients with advanced TC lose the ability to respond to RAI therapy and no effective alternative therapies are available. Defective NIS expression is the main reason for impaired iodide uptake in TC and NIS downregulation has been associated with several pathways linked to malignant transformation. NF-κB signaling is one of the pathways associated with TC. Interestingly, NIS expression can be negatively regulated by TNF-α, a bona fide activator of NF-κB with a central role in thyroid autoimmunity. This prompted us to clarify NF-kB’s role in this process. We confirmed that TNF-α leads to downregulation of TSH-induced NIS expression in non-neoplastic thyroid follicular cell-derived models. Notably, a similar effect was observed when NF-κB activation was triggered independently of ligand-receptor specificity, using phorbol-myristate-acetate (PMA). TNF-α and PMA downregulation of NIS expression was reverted when NF-κB-dependent transcription was blocked, demonstrating the requirement for NF-kB activity. Additionally, TNF-α and PMA were shown to have a negative impact on TSH-induced iodide uptake, consistent with the observed transcriptional downregulation of NIS. Our data support the involvement of NF-κB-directed transcription in the modulation of NIS expression, where up- or down-regulation of NIS depends on the combined output to NF-κB of several converging pathways. A better understanding of the mechanisms underlying NIS expression in the context of normal thyroid physiology may guide the development of pharmacological strategies to increase the efficiency of iodide uptake. Such strategies would be extremely useful in improving the response to RAI therapy in refractory-TC.

## Introduction

The well-differentiated thyroid carcinomas (DTCs) arise from thyroid follicular cells and represent the most frequent forms of thyroid cancer (TC), including the papillary thyroid cancer (PTC) and follicular thyroid cancer (FTC) subtypes [1].

The majority of DTCs are associated with a favorable prognosis. However, about 30% of patients with advanced forms of DTC become resistant to radioactive iodine (RAI) therapy, the standard treatment for metastatic disease [2]. The lack of efficient therapeutic options alternative to RAI makes the clinical management of these patients challenging, reducing the 10-year survival rate from approximately 90% to 10% [2,3]. The main reason for impaired iodide uptake in refractory-TC is the defective functional expression of the sodium iodide symporter (NIS) [4,5]. NIS belongs to the human solute carrier (SLC) family of transporters, is highly expressed at the basolateral membrane of thyroid follicular cells and is responsible for the active transport of iodide across the plasma membrane into thyroid follicles [6]. The primary regulator of NIS expression in thyroid gland is the thyroid stimulating hormone (TSH) [7,8]. TSH-induced accumulation of cyclic AMP leads to the binding of the PAX8 transcription factor to the NIS upstream enhancer (NUE) element on the NIS gene promoter, a primary requirement for the full activation of NIS expression [9,10]. Despite TSH-derived signaling being the key regulator of NIS expression in thyroid tissue, other signaling pathways may have an impact on this process. NIS expression levels and iodine uptake in DTC are reduced when compared to normal tissue [11,12] and this downregulation has been associated with the overactivation of several pathways linked to thyroid malignancy [13]. NF-κB signaling has been implicated in cancer-associated processes of several human malignancies, including thyroid cancer. Increased NF-kB activation has been described in PTC, FTC and anaplastic TC, as being associated with resistance to apoptosis and maintenance of the malignant phenotype [14–16]. Also, in previous studies, we have shown that overexpression of tumor-related RAC1b, a highly activated splice variant of the GTPase RAC1 [17,18], has a significant role in PTC tumorigenesis by inducing resistance to programmed cell death through NF-κB activation [19].

The NF-κB pathway is also responsible for controlling several aspects of cell growth and inflammation [20]. One major pathway responsible for NF-κB activation is the canonical NF-κB pathway, which involves preferentially the heterodimer p65/p50 and is triggered in response to numerous stimuli including pro-inflammatory cytokines such as tumor-necrosis-factor-α (TNF-α) and bacterial lipopolysaccharide (LPS) [20–22].

The understanding of the mechanisms underlying NIS expression in the perspective of normal thyroid physiology may guide the development of strategies to enhance the efficiency of iodide uptake, particularly in the neoplastic context. In fact, in addition to its role in TC, NF-κB has also been implicated in normal thyroid physiology, being necessary for normal thyroid structure development and function, and involved in the expression of several thyroid specific genes, such as PAX8, Tg, TTF-1, TPO and also NIS [23,24]. Indeed, it was shown that the p65 subunit of NF-κB acts in synergy with the transcription factor PAX8 for the promotion of NIS expression, as the result of LPS stimulation [25]. Conversely, despite being widely recognized as a potent activator of the canonical NF-κB pathway [26], TNF-α was shown to have a negative impact in NIS expression [27]. This is consistent with the observed reduction in RAI uptake in DTCs with concurrent thyroiditis [28], as TNF-α is a central factor in thyroid autoimmunity.

This led us to raise the question of whether this negative impact of TNF-α on NIS expression involves activation of NF-κB.

## Materials and methods

### Cell lines and culture conditions

The TSH-responsive cell lines PCCL3 and FRTL5, derived from Fischer rat’s thyroid follicular normal epithelium, were maintained in Coon’s F-12 modified liquid medium (Merck) supplemented with 5% fetal bovine serum (FBS) (Gibco), 10 μg/mL of insulin (SIGMA), 5 μg/mL of Apo-Transferrin (Apo-T) (SIGMA) and 0.1 mU/mL of TSH (SIGMA). Cells were maintained at 37°C in a 5% humidified CO2 environment and discarded after 20 passages. When appropriate, cells were cultured in starvation medium (F12 Coon’s Modification medium supplemented with 0.2% (v/v) FBS and 5 μg/mL of Apo-T) or TSH-medium (starvation medium supplemented with 1 mU/mL of TSH). Stimulation with TNF-α (10 ng/mL, SIGMA) or phorbol 12-myristate 13-acetate (PMA; 10 ng/mL; SIGMA) was performed (60 minutes to 24 hours) in serum-starved cells for 72h, subsequently subjected to TSH stimulation for 24 hours (TSH- medium). When applicable, cells were treated with the NF-kB selective inhibitor BMS-345541 (10 μM, SIGMA) for 6 hours.

### RNA extraction, cDNA synthesis and RT-qPCR

Total RNA was obtained from cultured cells using the ready-to-use reagent TripleXtractor (GRiSP Research Solutions) following manufacturer’s instructions. cDNA was synthetized from 2 μg of RNA using random primers and RevertAid Reverse Transcriptase (Thermo Scientific), following the manufacturer’s protocol.

NIS expression levels were quantified by SYBR Green based quantitative reverse transcription polymerase chain reaction (RT-qPCR) on the Light Cycler 480II (Roche), using Xpert Fast SYBR (Grisp). HPRT1 was used as endogenous control gene. NIS levels were normalized to endogenous control expression level. NIS normalized values were then expressed relative to that of a reference sample (pool of TSH stimulated rat cell lines). Expression values correspond to arbitrary units representing fold differences relative to the reference sample. Primers used (synthesized by NZytech, Portugal) were the following: NIS F (5’-TCCTCACAGGCCGTATCTCA) and NIS R (5’–GAAGGAACCCTGGAGGACAC); HPRT1 F (5’-GCTGAAGATTTGGAAAAGGTG) and HPRT1 R (5’-AATCCAGCAGGTCAGCAAAG). IKBα expression levels were quantified through the same method as NIS using IKBα the specific primers IKBαF (5’-TCCTCACAGGCCGTATCTCA) and IKBαR (5’-GACACGTGTGGCCGTTGTAG).

### HS-YFP–based iodide influx assay

Iodide influx was accessed in PCCL3 cells stably expressing the halide-sensitive yellow fluorescent protein (HS-YFP-H148Q/I152L - Y-PCCL3 cells) [29,30]. Cells were seeded in 8-well chamber slides (Ibidi) and serum-starved for 24h. Subsequently, cells were stimulated for 96h with 1 mU/mL TSH and then treated with TNF-α (10 ng/mL, 24h) or PMA (10 ng/mL, 24h). Cells were washed with PBS and then incubated for 15 min at 37°C with influx-solution (137 mM NaCl, 2.7 mM KCl, 0.7 mM CaCl2, 1.1 mM MgCl2, 1.5 mM KH2PO4, 8.1 mM Na2HPO4, and 10 mM glucose, pH 7.4). Each well was assayed individually for iodide influx by recording fluorescence continuously in a Leica TCS-SPE confocal microscope after addition of isomolar PBS-NaI solution (1 mM final NaI concentration). Decay of YFP fluorescence was followed for 600 seconds, acquiring an image every 10s. To confirm that the iodine influx observed was specifically mediated by NIS, additional assays were performed in the presence of ClO4- (a competitive inhibitor of iodide uptake by NIS), in which cells were incubated with 1mM ClO4-, for 10min, before PBS-NaI solution was added. Images were stacked and analyzed with Image J, defining whole field regions of interest (ROIs) for pixel intensity measurements that excluded saturated cells. Cell fluorescence recordings were normalized for the initial average value measured before addition of I−.

#### Total protein lysates and western blot

Y-PCCl3 cells were serum-starved for 24h, stimulated for 96h with 1 mU/mL TSH and then treated with TNF-α (10 ng/mL, 24h) or PMA (10 ng/mL, 24h). Protein extracts were prepared in Laemmli sample buffer (supplemented with 1 U/μl Benzonase, Sigma-Aldrich) and resolved, according to standard protocols, in 12% SDS-PAGE and transferred to PVDF membranes (Bio-Rad). The primary antibody rabbit polyclonal anti-NIS (Proteintech) was used in Western blot at 1:1000. Antibody mouse monoclonal anti-PCNA (Merck) was used at 1:10000. Detection was carried out using secondary peroxidase-conjugated anti-rabbit or anti-mouse IgG (Bio-Rad) antibodies followed by chemiluminescence.

### Data and statistical analysis

The data used to support the findings of this study are included within the article. Statistical analysis was carried out using GraphPad Prism statistical software (San Diego, CA). Quantitative results are shown as means ± SD of at least three independent observations. Statistical comparisons of data sets were made using unpaired two-tailed Student’s *t*-test and statistical significance was accepted when *p* values < 0.05.

In the halide-sensitive YFP-based functional assay, the signal decay caused by YFP fluorescence quenching was fitted to an exponential decay function to derive the maximal slope that corresponds to initial influx of I^−^ into the cells. Maximal slopes were converted into rates of variation of the intracellular I^−^ concentration (in nmol/min) using the equation d[I^−^]/dt = K_d_[d(F/F_0_)/dt], where K_d_ is the affinity constant of YFP for I^−^, and F/F_0_ is the ratio of the cell fluorescence at a given time versus the initial fluorescence [31,32].

## Results and discussion

TNF-α is a pro-inflammatory cytokine widely defined as a potent activator of the canonical NF-κB pathway. TNF-α has been previously reported to induce a negative impact on NIS expression by downregulating NIS mRNA levels up to 70% in FRTL5 rat thyroid cells [27]. However, whether this effect was dependent of NF-κB activation remained elusive.

NIS expression levels are typically reduced in malignant thyroid tissue relative to normal tissue [11,12]. This results from multiple mechanisms elicited by several signaling pathways involved in thyroid tumorigenesis [33]. Consistently, most of the DTC-derived cell lines available fail to express thyroid-specific genes, including NIS and the TSH receptor [34], which hampers the study of TSH-mediated regulation on NIS expression in these neoplastic cellular models (S1 Fig). Nevertheless, the study of the mechanisms underlying NIS functional regulation using non-neoplastic, TSH-responsive, thyroid cell models have been shown to allow the identification of key regulatory events that can be further translated into the malignant context [35–38].

**Fig 1:**
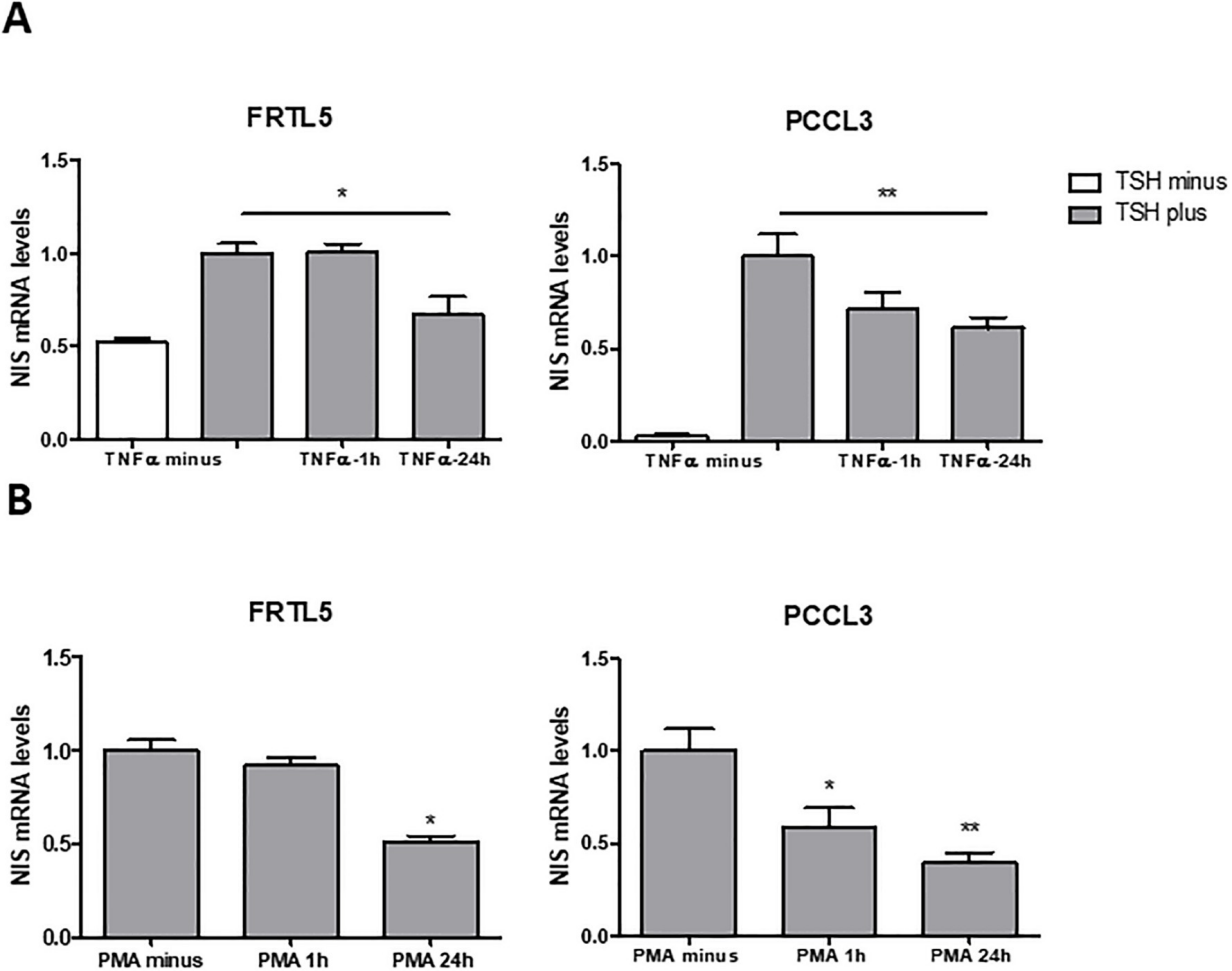
Effect of TNF-α and PMA on NIS transcriptional expression in non-neoplastic, TSH-responsive thyroid follicular cell lines. NIS mRNA levels were assessed by RT-qPCR and correspond to arbitrary units representing fold differences relative to a reference sample, corrected to HPRT levels used as endogenous control gene. Impact on NIS transcript levels of TNFα (A) and PMA (B) stimuli. FRTL5 and PCCL3 were subjected to a 96h starvation period followed by stimulation with TSH (1 mU/mL for 24h), in the presence of either TNFα (10 ng/mL; 1h and 24h) or PMA (10 ng/mL; 1h and 24h). As controls, non-stimulated cells and cells stimulated with TSH for 24h in the absence of TNFα and PMA were included. Plotted values are the mean ±SD (error bars) of three independent assays. Comparisons of TNF-α and PMA stimulation to non-stimulated conditions were made using two-tailed Student’s t-tests (*p≤0.05; **p ≤0.01).

In this study, we started by assessing TNF-α impact on TSH-induced NIS expression using the PCCL3 cell line as an additional cellular model. This, like the FRTL5 cell line, is also a non-neoplastic rat thyroid follicular cell-derived model representative of TSH-responsive system for NIS expression and iodide uptake [8]. Serum-starved cells were subjected to TSH stimulation and then treated with TNF-α for 1h or 24h. The effects on NIS mRNA levels were accessed by qRT-PCR (Fig 1A). We confirmed that TNF-α led to downregulation of TSH-induced NIS expression in FRLT5. This effect was also observed in PCCL3 cells, reaching a statistically significant decrease with a 24h treatment (Fig 1A). Next, we asked whether NIS downregulation was a specific outcome of TNF-α stimulation or if it may result from stimulation with other NF-κB activators. In fact, in contrast to that observed with TNF-α treatment, NIS levels were shown to increase upon stimulation of FRTL5 cells with LPS, another well-defined activator of the canonical NF-κB pathway. This increase was actually shown to be a result of the synergistic action of PAX8 and the p65-NF-κB subunit in the NUE regulatory region [25]. This suggests that different triggers for NF-κB activation may result in opposite NIS expression outcomes. These may be a consequence of the concerted action between the signaling cues generated by ligand-receptor interaction and the NF-κB active dimers produced. Thus, we then tested the effect of NF-κB activation on NIS expression when this is triggered independently of ligand-receptor specificity by stimulating the cells with phorbol-myristate-acetate (PMA). This has been shown to induce canonical NF-κB-dependent transcription by acting through the direct activation of protein-kinase-C [39]. We observed that, similarly to TNF-α, PMA downregulates NIS mRNA expression in both PCCL3 and FRTL5 cells (Fig 1B). It should also be noted that, albeit PMA has been shown to affect NIS protein acting as a downregulator of NIS function and iodine uptake in human breast cells [39,40], no effects at NIS transcriptional control level have been reported so far.

**Fig 2:**
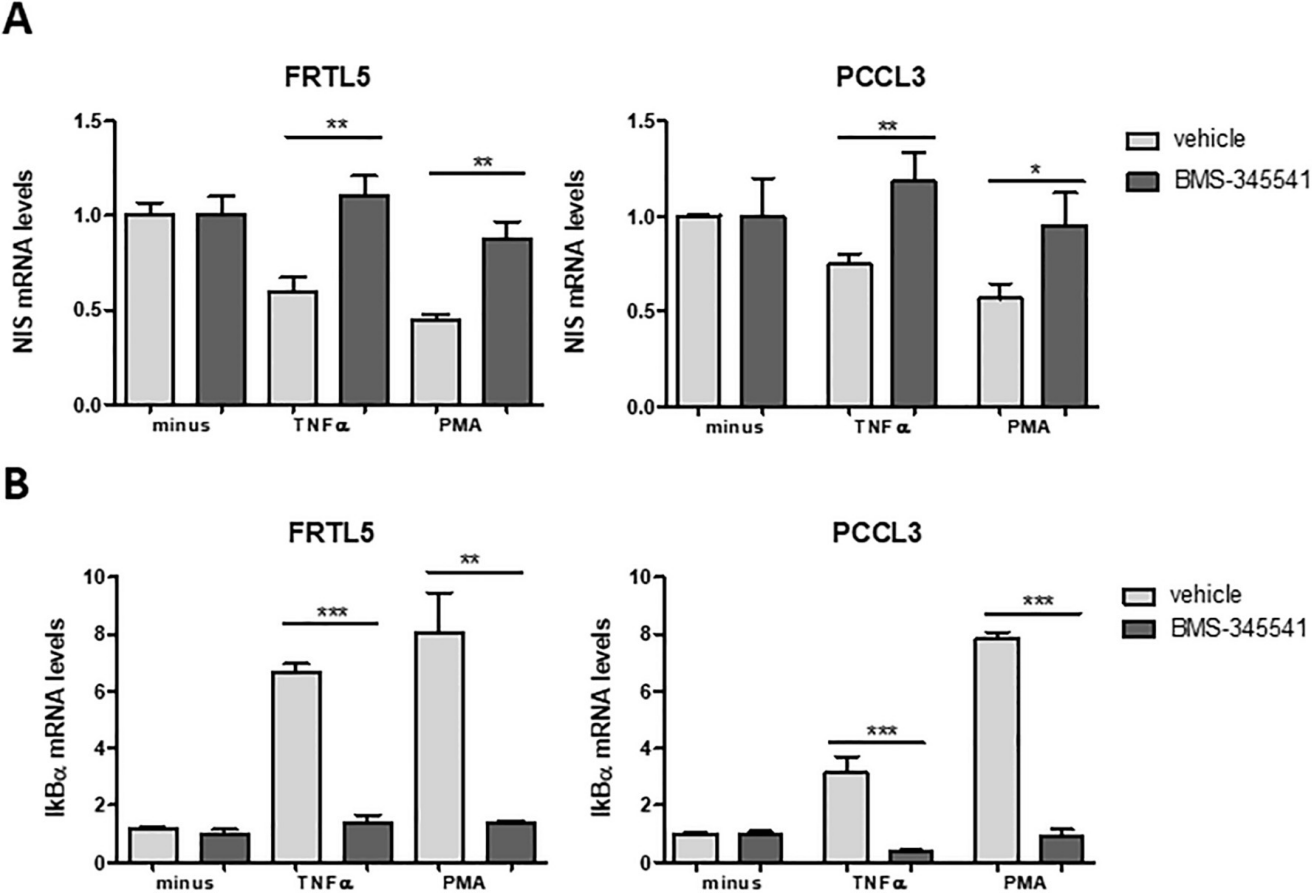
NF-κB impact on TNF-α- and PMA-mediated downregulation of NIS transcriptional expression. NIS (A) and IKBα (B) mRNA levels were quantified by RT-qPCR. TSH-stimulated cells were treated for 24h with TNF-α and PMA either in the absence (vehicle) or presence of the NF-κB inhibitor BMS-345541 (10 μM; 6h). Plotted values are the mean ±SD (error bars) of three independent assays, compared with the group treated with only TSH. Comparisons were made using two-tailed Student’s t-tests (*p≤0.05; **p ≤0.01; *** p ≤0.001).

Our next step was to clarify whether this negative impact on NIS expression was in fact dependent on the NF-κB-mediated transcriptional activity. Thus, we assessed the effect of TNF-α and PMA in the presence of BMS-345541, an allosteric inhibitor of the IkB Kinase (IKK) complex [41] whose activity is mandatory for canonical NF-κB activation [20]. We observed that, in the presence of BMS-345541, the decrease in NIS transcript levels induced by both TNF-α and PMA was reverted, with NIS mRNA reaching levels similar to those of TSH stimulation alone (Fig 2A). This observation is consistent with NF-κB activation being required for NIS downregulation.

NF-κB activation by TNF-α and PMA was further monitored using IκBα mRNA levels as a readout of NF-κB transcriptional activity. In fact, one of the early events resulting from canonical NF-κB activation is the increase of IκB-α mRNA levels [20] and it has been previously shown that IκBα mRNA levels correlate robustly with the activation of NF-κB in several cellular models, making it a broadly effective readout of NF-κB activation [42]. A significant increase in IκB-α mRNA levels was observed upon stimulation of both PCCL3 and FRTL5 cells with TNF-α and PMA. Moreover, this increase was strongly inhibited by BMS-345541, indicating a specific response from the NF-κB canonical pathway (Fig 2B).

**Fig 3:**
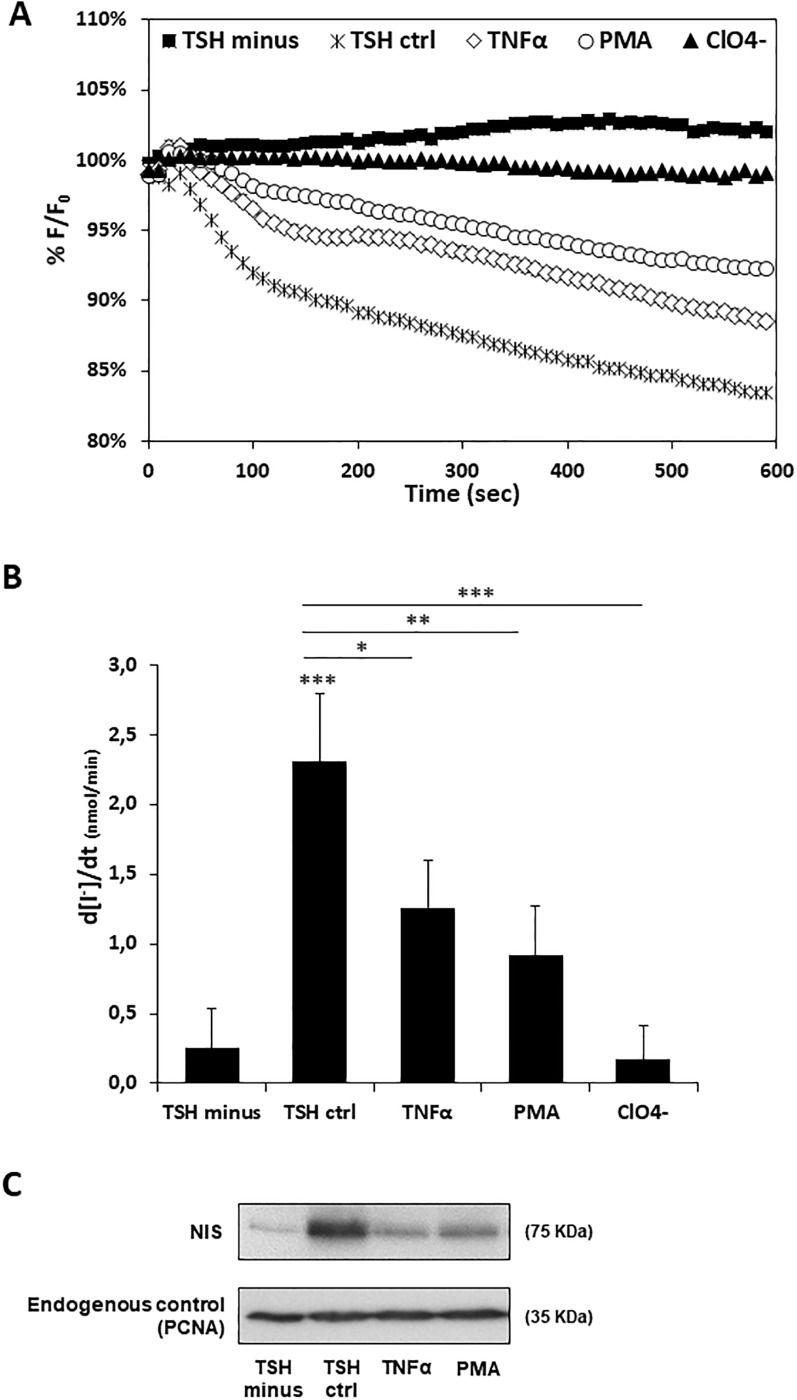
Effect of TNF-α and PMA on TSH-induced iodide uptake and NIS protein levels in Y-PCCL3 cells. Cells PCCL3 cells stably expressing the YFP-halide sensor were subjected to a 24h starvation period and then treated with TSH for 96h (TSH ctrl), in the presence or absence of either TNF-α or PMA Additional iodide influx assays were performed in the presence of both (24h treatment), and subjected to iodide influx assays and Western blot. TSH and ClO4- (1 mM, 10 min), a competitive inhibitor of iodide uptake by NIS. Representative HS-YFP fluorescence decay traces (A) recorded continuously for 600 seconds, acquiring an image every 10 s, after exposure to 1mM NaI (as described in [30]). Fluorescence (F) was plotted over time as percentage of fluorescence at time 0 (F0). Iodide influx rates (B) calculated by fitting the curves to the exponential decay function to derive the maximal slope that corresponds to initial influx of I^−^ into the cells. Endogenous NIS protein expression upon TSH, TNF-α or PMA treatment was monitored Western Blot using anti-NIS primary antibody. Endogenous PCNA expression was used as loading control. Data are means ± SEM of three independent assays. Comparisons were made using one-tailed Student’s t-tests (*p≤0.05; **p ≤0.01; ***p ≤0.001).

Finally, we asked whether TNF-α and PMA would also have a negative impact on iodide uptake. To address this question, we established an iodide influx assay in PCCL3 cells, modified to stably express the halide-sensitive yellow fluorescent protein (YFP) F46L/H148Q/I152L mutant (HS-YFP) [17,31,43] which has been validated for NIS activity assessment [29,30]. The HS-YFP inside these modified PCCL3 cells (Y-PCCL3) is quenched by iodide, enabling changes in iodide intracellular concentration to be monitored in thyroid cells by live cell imaging. Y-PCCL3 cells were thus subjected to TSH stimulation in the presence of TNF-α and PMA. A nine-fold faster decay rate of HS-YFP fluorescence was observed upon TSH stimulation, compared to that detected in the absence of TSH (Fig 3A and 3B), consistent with TSH enhancement of NIS expression that ultimately promotes NIS-mediated iodide uptake. We confirmed that the observed TSH-induced iodide influx was mediated by NIS since it was completely abolished in the presence of perchlorate (ClO4-) (Fig 3A and 3B), a competitive inhibitor of iodide uptake by NIS [44]. In addition, both TNF-α and PMA stimuli induced a meaningful decrease in TSH-induced iodide influx in Y-PCCL3 cells (Fig 3A and 3B). Consistent with the iodide influx rates and the observed downregulation of NIS expression at transcriptional level, a considerable reduction in NIS protein levels was also noted in the presence of both TNF-α and PMA (Fig 3C).

Transcriptional and post-transcriptional regulation of NIS expression involves different molecular players and signaling pathways that are also implicated in tumor progression and aggressiveness. NF-κB family of transcription factors is responsible for regulating many cellular processes such as inflammatory responses, cellular growth and cell death, and has been also implicated in TC development. We have previously reported NF-kB activation as a key mechanism through which the tumor-related RAC1b exerts its oncogenic action in the context of PTC [19]. Additionally, we have also recently shown an inverse correlation between RAC1b overexpression and NIS levels in DTCs [45]. This prompted us to explore, in the present study, the impact of the NF-κB signaling on NIS expression. Our data support that the canonical NF-κB pathway may also be involved in the downregulation of NIS.

The interplay between the NF-κB activation and NIS expression, however, requires further clarification. This is particularly important when we take into account that the effects on NIS transcription induced by TNF-α and PMA are opposite to those described for LPS [25]. One possible explanation for these apparent antagonistic effects of NF-κB activation in NIS transcription may be the activation and interplay of additional pathways that concertedly modulate the affinity of NF-κB transcription factors for different promoters. Thus, NF-κB-driven transcription may lead to different results depending on the overall signaling involved in its activation and regulation.

Understanding the mechanisms underlying physiological NIS functional regulation may allow the identification of regulatory events that can be pharmacological targeted to enhance the iodide uptake efficiency and improve the response to RAI therapy in RAI-refractory thyroid cancers.

## Supporting information

S1 Fig Effect of TSH on NIS transcriptional expression in thyroid follicular cell-derived cell lines. NIS mRNA levels were assessed by RT-qPCR and correspond to arbitrary units representing fold differences relative to a reference sample, corrected to endogenous control expression levels. HPRT1 and TBP were used as endogenous control genes for rat and human, respectively. RT-qPCR preformed as previously described [45]. The different cell lines were subjected to a 96h starvation period followed by stimulation with TSH (1 mU/mL for 48h). Plotted values are the mean ±SD (error bars) of three independent assays.
(TIF)
