# Supplementary figures and images for: TNFα-mediated activation of NF-κB downregulates sodium-iodide symporter expression in thyroid cells
Source: PLoS One. 2020 Feb 12;15(2):e0228794. doi: 10.1371/journal.pone.0228794 (PMC7015378; doi:10.1371/journal.pone.0228794)

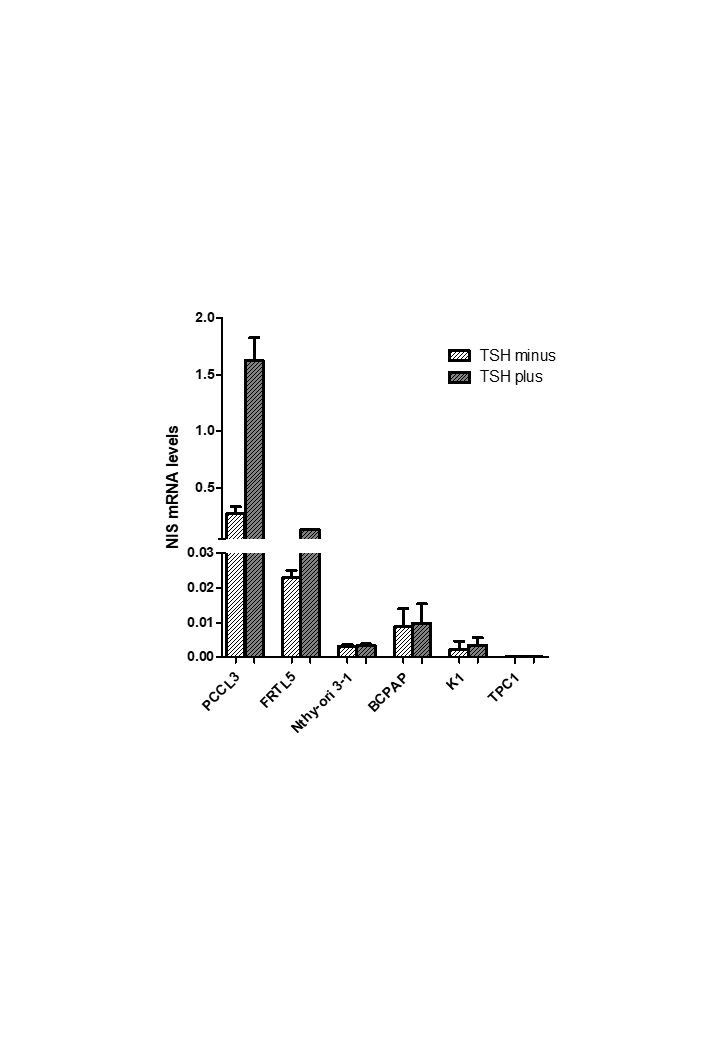

Supplement: S1 Fig — NIS mRNA levels were assessed by RT-qPCR and correspond to arbitrary units representing fold differences relative to a reference sample, corrected to endogenous control expression levels. HPRT1 and TBP were used as endogenous control genes for rat and human, respectively. RT-qPCR preformed as previously described [45]. The different cell lines were subjected to a 96h starvation period followed by stimulation with TSH (1 mU/mL for 48h). Plotted values are the mean ±SD (error bars) of three independent assays. (TIF) [file pone.0228794.s001.tif]
